# Supplementary material for: Effect of an Electronic Alert System on Hepatitis B Virus Reactivation in Patients Receiving Immunosuppressive Drug Therapy
Source: J Clin Med. 2022 Apr 26;11(9):2446. doi: 10.3390/jcm11092446 (PMC9104084; doi:10.3390/jcm11092446)
Supplement: Supplementary file 1 [file jcm-11-02446-s001.zip › jcm-1652752-supplementary.pdf]

Supplemental Table S1. Correlation of HBsAg-positive (>12.65 IU/mL) and HBV-DNA-positive status.

	HBV-DNA (+)	HBV-DNA (-)
HBsAg (>12.65 IU/mL)	21	8
HBsAg (≤12.65 IU/mL)	4	10

Abbreviations: HBV, Hepatitis B virus; HBsAg, hepatitis B surface antigen.
